# MDA5-positive dermatomyositis: an uncommon entity in Europe with variable clinical presentations

**DOI:** 10.1186/s12948-015-0031-y

**Published:** 2015-11-09

**Authors:** Paola Parronchi, Anna Radice, Boaz Palterer, Francesco Liotta, Cristina Scaletti

**Affiliations:** Unit of Internal Medicine, Department of Experimental and Clinical Medicine, University of Florence, Largo Brambilla 3, 50134 Florence, Italy

**Keywords:** Dermatomyositis, MDA5, CADM, Clinically amyopathic dermatomyositis

## Abstract

Clinically amyopathic dermatomyositis (CADM), described almost 50 years ago, is defined on the basis of still not validated criteria and characterized by skin findings almost without muscle weakness. Autoantibodies directed against the cytosolic pathogen sensor MDA5 (CADM 140) can mark this subtype of dermatomyositis which has been reported to associate, in particular ethnic groups, with severe progressive interstitial lung disease, poor prognosis and an hyperferritinemic status resembling hemophagocytic-like syndromes. MDA5 may be relevant in that Interferon-signature claimed to characterize inflammatory myopathies and dermatomyosits itself, but its role is not clear. However, the titre of anti-MDA5 autoantibodies seems to correlate with the outcome. In Caucasian populations the association between anti-MDA5 positive CADM and rapidly progressive interstitial lung disease seems to be weaker, but the limited numbers of patients described so far could explain the lack of statistical significance. As a fact, European patients with circulating anti-MDA5 autoantibodies may be clinically inhomogeneous and exhibit different rates of severity. The two patients affected by anti-MDA5 positive dermatomyositis described hereafter provide a clear example of the extreme variability of the disease in terms of laboratory findings and clinical features.

## Background

Myositis-specific autoantibodies (MSAs) are closely associated with Dermatomyositis (DM), a rare systemic autoimmune disease characterized by skin involvement and muscle inflammation of variable entity. MSAs are mutually exclusive and differently related to clinical manifestations, complications, response to therapy and prognosis [1, 2]. Among them, anti-MDA5 autoantibodies have been associated with a subtype of DM with scarce muscle inflammation, classical skin disease and highly variable systemic manifestations.

We report here two cases of DM recently observed in our Department who confirm the high variability in the clinical presentation when circulating anti-MDA5 autoantibodies are present.

## Case reports

### Patient #1

**Fig. 1:**
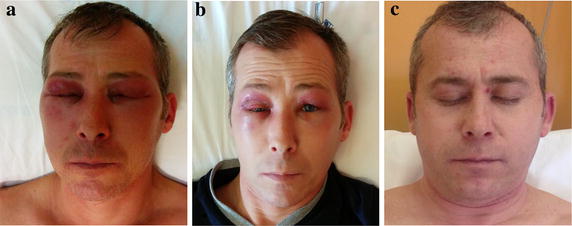
Severe involvement of eyelids at the admission into the Dept. (**a**), after pulse therapy with steroids (**b**) and during treatment with IVIg, Cyclosporine A and hydroxychloroquine (**c**)

**Fig. 2:**
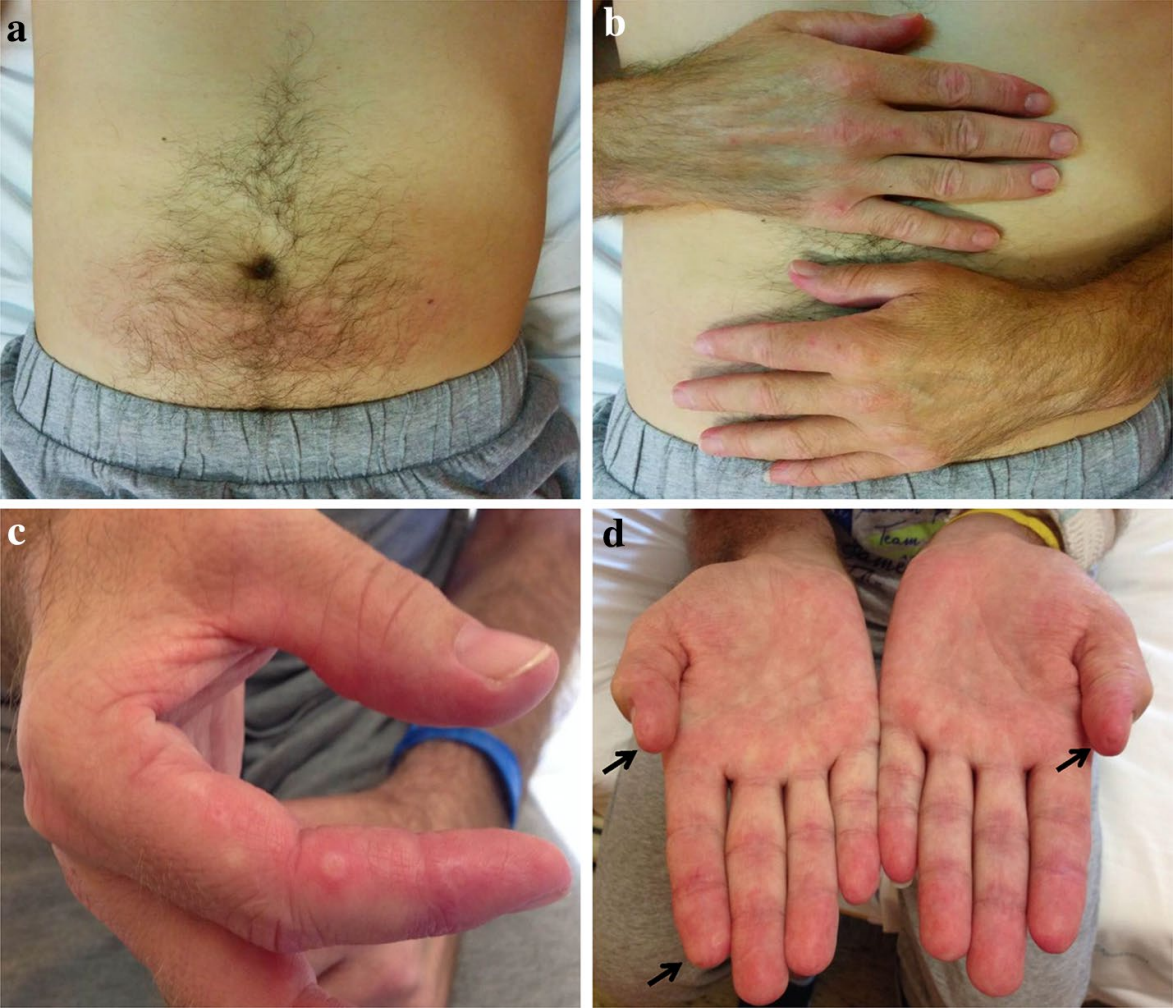
Violaceous erythema of the abdomen (**a**), Gottron’s sign (**b**), tender palm papules (**c**) with signs of necrosis (**d** *arrows*) of patient #1 at admission

**Fig. 3:**
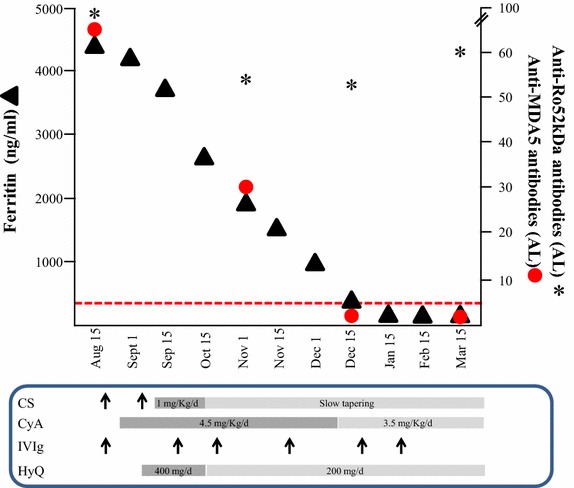
Disease activity and its relationship with autoantibodies. The graphic shows the relationship between ferritin values (ng/ml *black triangles*) as index of disease activity, levels of anti-MDA5 antibodies (*red dots*) and anti-Ro52 kDa (*black asterisks* both expressed as Arbitrary Levels, AL), as determined by a commercial immunoblot, and therapeutic interventions in patient #1. Normal values of ferritin are below 272 ng/ml (*red dotted line*), levels of autoantibodies are considered as negative below 10, as indicated by the manufacturer (Euroimmun AG). *CS* corticosteroids (methilprednisolone, prednisone); *CyA* cyclosporine A; *IVIg* immunoglobulins; *HyQ* hydroxychloroquine. *Upward pointing arrow* indicates iv pulse therapy

A 38 years old man from East Europe was admitted in our Dept. because of severe heliotrope rash with impressive eyelid oedema (Fig. 1a), mouth ulcerations, Gottron’s papules and diffuse Gottron’s sign (Fig. 2a, b), tender palmar papules with signs of necrosis (Fig. 2c, d). Within 2 days the patient developed severe hypoxemia (pO_2_ 48 mmHg) and a severe reduction in DLCo (42 %) associated with radiographic features of alveolitis and ground glass, in addition to intense muscle weakness and dysphagia. He also started to exhibit high grade continuous fever (>39 °C) insensitive to ordinary antipyretics while the presence of any infectious agent was excluded. Severe leucopenia together with anemia and thrombocytopenia, increased muscle and liver enzymes, hyperferritinemia were demonstrated (Table 1) while electromyography reported only mild myositis. Antinuclear autoantibodies were positive along with anti-MDA5 in association with anti-Ro52. Presence of malignancies was excluded. The diagnosis of scarcely myopathic but highly aggressive dermatomyositis was thus formulated. High dose steroids (iv pulse therapy with 1 gr 6MP for 5 days with slow oral taper-out) followed by IVIG (2 g/kg/d over 3 days with a second reload after 21 days), oral Cyclosporin A (4 mg/kg/d) together with hydroxychloroquine (5.4 mg/kg/d) were used as therapy with stunning results. Heliotrope rash rapidly improved and facial oedema reduced as well (Fig. 1b, c). O_2_ arterial pressure also increased (pO_2_ 86 mmHg, expected age-related value 92 ± 4 mmHg) and amelioration of lung function (DLCo 86 %) was observed whereas normalization of ferritin and liver enzymes was obtained in a two-months span together with disappearance of anti-MDA5 antibodies. The relationship between ferritin values, considered as the best indicator of disease activity, levels of anti-MDA5 (and anti-Ro52 kDa, as control) autoantibodies and therapeutic interventions are depicted in Fig. 3.

**Table 1:** Comparison between the laboratory findings in the two MDA5+ patients with dermatomyositis

	Patient #1	Patient #2	Reference values
Total blood counts			
WBC	1.67	5.17	4–10 × 10^9^/L
Neutrophils	1.08	1.66	1.5–7.5 × 10^9^/L
Lymphocytes	0.27	2.67	1–5 × 10^9^/L
Hb	9.8	12.8	13–16 g/dL
PLTs	130	217	140–440 × 10^9^/L
Liver enzymes			
AST	244	28	15–37 U/L
ALT	263	48	12–65 U/L
Cholestasis indexes			
ɣ-GT	399	117	5–85 U/L
AP	120	92	50–136 U/L
Total bilirubin	0.7	0.5	0.2–1 mg/dL
Muscle enzymes			
LDH	483	255	84–246 U/L
CPK	662	28	35–232 U/L
Aldolase	11.2	7.4	<7.3 U/mL
Indexes of inflammation			
ESR	50	41	2–10 mm/h
CRP	<9	<9	<9 mg/L
Ferritin	4218	292	26–388 ng/mL
Autoantibodies			
ANA	1/640	Neg	<1/80
Anti-MDA5	+++ (72)	+++ (58)	Neg
Anti-Ro52	+++ (84)	Neg	Neg

All the values reported were measured at admission in the absence of therapy. Anti-nuclear antibodies (ANA) were determined by means of indirect immunofluorescence on Hep2 cells (Euroimmun). Anti-MDA5 and anti-Ro52 kDa were determined by a validated commercial Immunoblot method (Euroimmun) (in brackets, levels of autoantibodies as automatically calculated by the EUROLineScan software)

### Patient #2

A 60 years old Italian woman was admitted in our Dept. because of heliotrope rash, Gottron’s sign and papules, shawl sign, periungueal teleangectasias, mechanic’s hands and subcutaneous calcinosis appeared 2 months before. She complained from worsening arthralgias (hands, wrists, feet) and generalized asthenia but without frank muscular pain or weakness. Electromyography showed mild myopathic signs. Spirometry, CO diffusion, arterial blood gas analysis and chest HRCT were unremarkable showing no sign of interstitial lung disease. Further exams excluded a paraneoplastic manifestation. Antinuclear antibodies were negative but intense positivity of anti-MDA5 antibodies was found. Liver and muscle enzymes as well as numbers of blood cells and ferritin levels were normal (Table 1), whereas ESR was mildly increased (Table 1). The diagnosis of amyopathic dermatomyositis was formulated and treatment with high dose IVIG (2 g/kg/d over 3 days) and methylprednisolone (60 mg with slow tapering) followed by azathioprine (1 mg/kg/d) was started rapidly achieving full control of the manifestations and disappearance of MDA5 autoantibodies.

## The intracellular sensor of foreign nucleic acids and interferon-inducer MDA5 and its role in autoimmunity

Melanoma-differentiation-associated gene 5 (MDA5), also known as Ifih1 or Helicard, discovered by Andrejeva et al. [3]. in 2004, is as an intracellular pathogen sensor located in the cytosol and belonging to the family of RIG-I-like receptors (RLRs) as well as RIG-I [4]. In physiological conditions, it binds long-sized (>1000 bp) viral double-stranded RNA without any end-specificity whereas Poly (I:C) represents its synthetic activator [5]. Similarly to RIG-I, MDA5 consists in two N-terminal caspase recruitment domains (CARDs), two DExD/H-box helicase domains and a C-terminal domain (CTD). The two helicase domains wrap around dsRNA and CTD contacts one of them with the formation of a sort of a closed ring around the RNA [6, 7]. MDA5 is thightly regulated by ATP, as binding induces assembly and hydrolysis causes depolymerization, and LGP2, a third RLR-member, as able to contact dsRNA but unable to signal as CARD-lacking [8, 9]. MDA5 assembly, partially dependent from Lys63- linked ubiquitin, activates mithocondrial antiviral signalling protein MAVS (also known as CARDIF or VISA or IPS-1) situated on the mitochondrial and peroxisomal membranes. At least 11 MDA5 molecules are necessary to recruit MAVS. CARD (on MDA5)-CARD (on MAVS) interaction induces polymerization of MAVS which switches from a soluble form into a self-propagating helical fiber [10]. This likely remodels organelle membranes and activates the expression of several anti-viral defense factors including Interferons (IFNs). Along with this, binding of MDA5 to MAVS also activates the cytosolic protein-kinases Ikk and TANK-binding kinase 1 (TBK1) which, in turn, activate transcription factors NF-kB, IRF3 and IRF7 [11]. These factors translocate into the nucleus inducing the expression of several proteins including MDA5 itself, thus creating an amplifying inflammatory loop. Physiologically, RLRs are key protectors from RNA viruses, are expressed in different cell types (dendritic cells, epithelial cells and fibroblasts) and are involved in the production of IFN-alfa in addition to pro-inflammatory chemokines and cytokines [6, 12].

Overproduction (or abnormal production at certain sites) of IFN-alfa has been claimed to be the signature of autoimmune diseases (so called ‘IFN-alfa signature’) such as systemic lupus erythematosus (SLE), type I diabetes and myositis [13–15]. Recognition of foreign or host DNA indeed induces IFN-alfa production via Toll-like receptor (TLR) 9 and/or cytosolic DExD/H-box helicase-containing sensors, but these latter do not include RLRs. Thus, the role exerted by MDA5 should be different, i.e. altered conformation leading to increased type I IFN expression. Along with this, functional genetic polymorphisms of MDA5 gene (A946T) associated with overactivity have been found in SLE [16, 17] and the susceptibility to a systemic autoimmune diseases resembling SLE has been recently confirmed in an experimental model of MDA5 single coding-change mutation [18]. Secondly, Thr946/Thr946 homozygous individuals are at risk of type I diabetes. However, as this gene allelic variation is not pathogenic ‘per se’, alterations in negative regulatory genes may be probably necessary for overt autoimmune diseases [17]. GWAS studies found MDA5 polymorphisms in Rheumatoid Arthritis and Multiple Sclerosis, nonetheless loss-of-function mutations would be protective against these diseases [19]. Further, gain-of-function mutations of MDA5 were identified in the monogenic interferonopathy Aicardi-Goutières Syndrome (AGS) and neuro-inflammatory phenotypes characterized by an IFN-alfa signature. These mutations increase basal signalling because of the increased affinity to dsRNA or the impaired hydrolysis by ATP, even in the absence of foreign RNA [19].

Finally, even if MDA5 overreactivity, followed by increased production of IFN-alfa, can only be speculated in DM and mechanisms are still undisclosed, it is indeed true that titers of anti-MDA5 autoantibodies are directly related with disease activity and worse prognosis whereas their disappearance indicates disease control thanks to therapy [20]. However, it is not clear whether these autoantibodies represent a simple epiphenomenon or act as agonists on the immune sensor or initiators of cytotoxicity. In addition, although the role of type I IFNs and certain RLRs, such as RIG-I, have been recently confirmed in classic DM as found to be expressed at higher levels in muscle biopsies and human myotube cultures [21], clinical results from sifalimumab (anti-IFN-alfa mAb) need to be confirmed in larger trials as well as circulating IFN-alfa as a potential biomarker in MDA5-positive dermatomyositis need to be assessed by adequate protocols [22].

## Clinical characteristics of MDA5-positive dermatomyositis

DM is a rare autoimmune disease characterized by skin involvement, striate muscle inflammation which may include the upper oesophagus and possible damage of internal organs frequently marked by the presence of autoantibodies [23]. Several autoantibodies have been described in inflammatory myopathies and distinguished into myosytis-associated (MAA) and myositis-specific antibodies (MSA) [1] with recent novelties in this field. In particular, cortactin has been recognized as a new target antigen in polymyositis and immune-mediated necrotizing myopathies [24] along with anti-nuclear matrix protein 2/MJ (NXP2), associated with calcinosis [25] and, possibly, ILD [26]. The p155 recognized by anti-TIF-1ɣ and the small ubiquitin like modifier activating enzyme heterodimer eliciting anti-SAE have been related to malignancy in adults and severe course of DM, respectively [1, 25, 27]. Finally, anti-MDA5 (also known as anti-CADM140) were claimed to mark a DM variant with low grade/absent muscle inflammation (amyiopathic dermatomyositis) but severe skin manifestations, hyperferritinemic status, frequent and rapidly progressive interstitial lung disease (ILD) and poor prognosis [28–31].

The term of clinically amyiopathic dermatomyositis (CADM) was coined by Pearson [32] almost 50 years ago describing those 2–20 % DM patients with skin findings but without muscle weakness. In the early nineties classification criteria of CADM were proposed but never validated [33] and association with cancer and ILD was found. In a retrospective study from United States CADM appeared to mostly be a favourable disease [34] but soon after a study reviewing 301 patients underlined an unusual frequence of lung involvement (13 %). Interestingly enough, 111 patients in this cohort came from Eastern Asia [35].

In addition to heliotrope rash, Gottron and shawl sign, skin (and mucosa) involvement actually covers a wide spectrum of manifestations, including papules (frequently tender on palm), plaques, nodules and ulcerations. Painful ulcers usually localize on extensor surface of joints (fingers, elbow, knees), lateral nailfolds or digital pulp [36]. They can be also present in classic DM (usually below 20 %) but more frequently associate with malignancy, resistance to immunosuppressive therapy or, in Asian populations, lung disease [30]. A recent retrospective study partially confirms these findings as ulcers did not significantly associate with myopathy or other classical clinical features of DM (dysphagia, Raynaud’s or joint pain) but more commonly affected Asian patients. On the other hand, ulcers of finger pulp and nailfolds were more frequent in patients with anti-MDA5 autoantibodies and/or anti-Ro52 kDa [36, 37].

Hyperferritinemia can be a primary condition (hemochromatosis, ferritin gene mutations), expression of primary or acquired hemophagocytic lymphohistiocytosis (HLH) or associated with inflammatory, infectious or malignant disorders. In autoimmunity, hemophagocytic-like syndromes or macrophage activation syndromes (MAS) can occur in lupus erythematosus, systemic onset of juvenile arthritis, catastrophic anti-phospholipid syndrome and adult onset Still’s disease [38]. MAS have been rarely described in the setting of DM, both classic and amyopathic one and are characterized by a septic-like spectrum with high fever, hepato-splenomegaly, lymphoadenopathies, cytopenias and increased levels of transaminases [39, 40]. In the Japanese series of CADM patients with circulating anti-MDA5 antibodies, hyperferritinemia was associated with severe ILD and fatal outcome with an estimated cut-off as predictor of death >1500 ng/ml [31, 41].

The interplay between CADM and interstitial lung disease is more intriguing. In the early 2000, several case reports from Japan, China and Korea suggested that CADM was someway related to severe ILD, rapid onset of respiratory failure and death within few months from the diagnosis (2–6 mo). Depending from the studies, 30–60 % of CADM Asian patients had ILD and the 6-month survival rate was about 40 % despite adequate treatment with high dose steroids and immunosuppressants [29, 42]. As a fact, in 2005 Sato et al. [30] had already described Japanese patients with CADM and severe ILD as showing circulating anti-CADM140 antibodies and 4 years later the same group identified MDA5 as the target antigen [43]. However, ethnicity seems to play a fundamental role as suggested by the relationship between MDA5 positivity and ILD [29, 44]. Actually, aggressive course of DM in the lung has not been confirmed by American authors [37].

The demonstration of anti-MDA5 may still represent a challenge as the first commercially available immunoblot assay was only recently introduced into the market in the context of other myositis-specific target antigens. Immunoprecipitation using ^35^S and extractive MDA5 originally used by Japanese authors [30] has been replaced by ELISA assays set up by the use of recombinant molecules from a home-made cDNA library [20] or a commercial source [45] for coating. The same commercial recombinant MDA5 has been also applied in a self-made immunoblot [45]. As a fact, no extensive study comparing the efficiency of the different methods to detect anti-MDA5 has been produced so far. Anyhow, in the last 2 years, DM cases with circulating anti-MDA5 antibodies have been also described in populations of non-Asian descent. Three studies regarding epidemiology and clinical features of anti-MDA5+ DM in European Caucasian patients have been published so far. In the unique retrospective Italian study of Ceribelli et al. [46], anti-MDA5 antibodies were found in 5 of 34 consecutive patients with DM (15 %). This seems to be slightly above the percentages found in American studies (6 and 7 %, respectively) [36, 37]. In a Hungarian cohort of 337 patients with idiopathic inflammatory myopathies including DM, anti-MDA5 antibodies were never found [25]. On the other hand, and confirming the Italian data, in a large number of DM patients from the Mediterranean area [45], the presence of anti-MDA5 was found in 12 %, half of them with amyopathic disease. In this study, more than 50 % of the patients suffered from rapidly progressive ILD and showed significant shorter survival rate in addition to higher frequency of panniculitis. Interestingly enough and similarly to our patient #1, the Spanish Authors found that most of the MDA5+ patients with DM and severe ILD also showed circulating anti-Ro52 autoantibodies in an association which has been only recently recognized. Viceversa, probably due to the very low number of MDA5+ patients in the Italian study, no statistical differences in the severity of skin involvement or mortality was found in MDA5+ in comparison with non-MDA5 DM patients and the frequency of ILD itself was statistically borderline (p 0.048). It was indeed confirmed that MDA5+ patients were more frequently amyopathic (p < 0.001) but, again, no differences could be demonstrated in digit pulp necrosis, Raynaud’s phenomenon or joint pain between the two groups.

## Conclusions

The pattern of circulating myositis-specific autoantibodies in Dermatomyositis can provide useful information for systemic complications and prognosis of the disease even though their precise role in the pathogenesis need to be further characterized. Anti-MDA5 autoantibodies mark a subtype of DM of high severity in Asians whereas a highly variable clinical presentation is seen in the Caucasian population. In this latter, however, only a comparison between larger numbers of MDA5+ and non-MDA5 DM patients might confirm whether the spectrum of the disease is really related to ethnicity.

## Consent section

Written informed consent was obtained from the patient for publication of this case report and any accompanying images. A copy of the written consent is available for review by the Editor-in-Chief of the journal.

## Abbreviations

MSA: myositis specific antibodies
MAA: myositis associated antibodies
DM: dermatomyositis
6MP: 6-methylprednisolone
IVIG: intravenous immunoglobulins
CADM: clinically amyopathic dermatomyositis
ILD: interstitial lung disease

## References

[1] Tansley SL, McHugh NJ (2014). Myositis specific and associated autoantibodies in the diagnosis and management of juvenile and adult idiopathic inflammatory myopathies. Curr Rheumatol Rep.

[2] Gunawardena H, Betteridge ZE, McHugh NJ (2009). Myositis-specific autoantibodies: their clinical and pathogenic significance in disease expression. Rheumatology (Oxford).

[3] Andrejeva J, Childs KS, Young DF, Carlos TS, Stock N, Goodbourn S, Randall RE (2004). The V proteins of paramyxoviruses bind the IFN-inducible RNA helicase, mda-5, and inhibit its activation of the IFN-beta promoter. Proc Natl Acad Sci USA.

[4] Unterholzner L, Keating SE, Baran M, Horan KA, Jensen SB, Sharma S, Sirois CM, Jin T, Latz E, Xiao TS, Fitzgerald KA, Paludan SR, Bowie AG (2010). IFI16 is an innate immune sensor for intracellular DNA. Nat Immunol.

[5] Kato H, Takeuchi O, Sato S, Yoneyama M, Yamamoto M, Matsui K, Uematsu S, Jung A, Kawai T, Ishii KJ, Yamaguchi O, Otsu K, Tsujimura T, Koh C-S, Reis e Sousa C, Matsuura Y, Fujita T, Akira S (2006). Differential roles of MDA5 and RIG-I helicases in the recognition of RNA viruses. Nature.

[6] Reikine S, Nguyen JB, Modis Y (2014). Pattern Recognition and Signaling Mechanisms of RIG-I and MDA5. Front Immunol.

[7] Berke IC, Modis Y (2012). MDA5 cooperatively forms dimers and ATP-sensitive filaments upon binding double-stranded RNA. EMBO J.

[8] Takahasi K, Kumeta H, Tsuduki N, Narita R, Shigemoto T, Hirai R, Yoneyama M, Horiuchi M, Ogura K, Fujita T, Inagaki F (2009). Solution structures of cytosolic RNA sensor MDA5 and LGP2 C-terminal domains: identification of the RNA recognition loop in RIG-I-like receptors. J Biol Chem.

[9] Jiang X, Kinch LN, Brautigam CA, Chen X, Du F, Grishin NV, Chen ZJ (2012). Ubiquitin-induced oligomerization of the RNA sensors RIG-I and MDA5 activates antiviral innate immune response. Immunity.

[10] Chiang JJ, Davis ME, Gack MU (2014). Regulation of RIG-I-like receptor signaling by host and viral proteins. Cytokine Growth Factor Rev.

[11] Wu B, Peisley A, Richards C, Yao H, Zeng X, Lin C, Chu F, Walz T, Hur S (2013). Structural basis for dsRNA recognition, filament formation, and antiviral signal activation by MDA5. Cell.

[12] Unterholzner L (2013). The interferon response to intracellular DNA: why so many receptors?. Immunobiology.

[13] Niewold TB (2011). Interferon alpha as a primary pathogenic factor in human lupus. J Interferon Cytokine Res.

[14] Oliveira L, Sinicato NA, Postal M, Appenzeller S, Niewold TB (2014). Dysregulation of antiviral helicase pathways in systemic lupus erythematosus. Front Genet.

[15] Shrivastav M, Niewold TB (2013). Nucleic Acid sensors and type I interferon production in systemic lupus erythematosus. Front Immunol.

[16] Harley JB, Alarcón-Riquelme ME, Criswell LA, Jacob CO, Kimberly RP, Moser KL, Tsao BP, Vyse TJ, Langefeld CD, Nath SK, Guthridge JM, Cobb BL, Mirel DB, Marion MC, Williams AH, Divers J, Wang W, Frank SG, Namjou B, Gabriel SB, Lee AT, Gregersen PK, Behrens TW, Taylor KE, Fernando M, Zidovetzki R, Gaffney PM, Edberg JC, Rioux JD, Ojwang JO (2008). Genome-wide association scan in women with systemic lupus erythematosus identifies susceptibility variants in ITGAM, PXK, KIAA1542 and other loci. Nat Genet.

[17] Molineros JE, Maiti AK, Sun C, Looger LL, Han S, Kim-Howard X, Glenn S, Adler A, Kelly JA, Niewold TB, Gilkeson GS, Brown EE, Alarcón GS, Edberg JC, Petri M, Ramsey-Goldman R, Reveille JD, Vilá LM, Freedman BI, Tsao BP, Criswell LA, Jacob CO, Moore JH, Vyse TJ, Langefeld CL, Guthridge JM, Gaffney PM, Moser KL, Scofield RH, Alarcón-Riquelme ME (2013). Admixture mapping in lupus identifies multiple functional variants within IFIH1 associated with apoptosis, inflammation, and autoantibody production. PLoS Genet.

[18] Funabiki M, Kato H, Miyachi Y, Toki H, Motegi H, Inoue M, Minowa O, Yoshida A, Deguchi K, Sato H, Ito S, Shiroishi T, Takeyasu K, Noda T, Fujita T (2014). Autoimmune disorders associated with gain of function of the intracellular sensor MDA5. Immunity.

[19] Del Toro Duany Y, Wu B, Hur S (2015). MDA5—filament, dynamics and disease. Curr Opin Virol.

[20] Sato S, Kuwana M, Fujita T, Suzuki Y (2013). Anti-CADM-140/MDA5 autoantibody titer correlates with disease activity and predicts disease outcome in patients with dermatomyositis and rapidly progressive interstitial lung disease. Mod Rheumatol.

[21] Suárez-Calvet X, Gallardo E, Nogales-Gadea G, Querol L, Navas M, Díaz-Manera J, Rojas-Garcia R, Illa I (2014). Altered RIG-I/DDX58-mediated innate immunity in dermatomyositis. J Pathol.

[22] Horai Y, Koga T, Fujikawa K, Takatani A, Nishino A, Nakashima Y, Suzuki T, Kawashiri S-Y, Iwamoto N, Ichinose K, Tamai M, Nakamura H, Ida H, Kakugawa T, Sakamoto N, Ishimatsu Y, Mukae H, Hamaguchi Y, Fujimoto M, Kuwana M, Origuchi T, Kohno S, Kawakami A (2015). Serum interferon-α is a useful biomarker in patients with anti-melanoma differentiation-associated gene 5 (MDA5) antibody-positive dermatomyositis. Mod Rheumatol.

[23] Bohan A, Peter JB (1975). Polymyositis and dermatomyositis (first of two parts). N Engl J Med.

[24] Labrador-Horrillo M, Martínez MA, Selva-O’Callaghan A, Trallero-Araguás E, Grau-Junyent JM, Vilardell-Tarrés M, Juarez C (2014). Identification of a novel myositis-associated antibody directed against cortactin. Autoimmun Rev.

[25] Bodoki L, Nagy-Vincze M, Griger Z, Betteridge Z, Szöllősi L, Dankó K (2014). Four dermatomyositis-specific autoantibodies-anti-TIF1γ, anti-NXP2, anti-SAE and anti-MDA5-in adult and juvenile patients with idiopathic inflammatory myopathies in a Hungarian cohort. Autoimmun Rev.

[26] Gossez M, Levesque M, Khouatra C, Cottin V, Garnier L, Fabien N (2015). Interstitial lung disease in an adult patient with dermatomyositis and anti-NXP2 autoantibody. Eur Respir Rev.

[27] Tansley SL, Betteridge ZE, McHugh NJ (2013). The diagnostic utility of autoantibodies in adult and juvenile myositis. Curr Opin Rheumatol.

[28] Mammen AL (2010). Dermatomyositis and polymyositis: clinical presentation, autoantibodies, and pathogenesis. Ann N Y Acad Sci.

[29] Sato S, Kuwana M (2010). Clinically amyopathic dermatomyositis. Curr Opin Rheumatol.

[30] Sato S, Hirakata M, Kuwana M, Suwa A, Inada S, Mimori T, Nishikawa T, Oddis CV, Ikeda Y (2005). Autoantibodies to a 140-kd polypeptide, CADM-140, in Japanese patients with clinically amyopathic dermatomyositis. Arthritis Rheum.

[31] Gono T, Kawaguchi Y, Hara M, Masuda I, Katsumata Y, Shinozaki M, Ota Y, Ozeki E, Yamanaka H (2010). Increased ferritin predicts development and severity of acute interstitial lung disease as a complication of dermatomyositis. Rheumatology (Oxford).

[32] Pearson CM. Polymyositis and dermatomyosits. In: Hollander JL, McCarty DJ, editors. Arthritis and allied conditions: a textbook of rheumatology. 8th ed. Philadelphia: Lea & Febiger; 1972. p. 940–61.

[33] Sontheimer RD (2002). Would a new name hasten the acceptance of amyopathic dermatomyositis (dermatomyositis siné myositis) as a distinctive subset within the idiopathic inflammatory dermatomyopathies spectrum of clinical illness?. J Am Acad Dermatol.

[34] el-Azhary RA, Pakzad SY (2002). Amyopathic dermatomyositis: retrospective review of 37 cases. J Am Acad Dermatol.

[35] Gerami P, Schope JM, McDonald L, Walling HW, Sontheimer RD (2006). A systematic review of adult-onset clinically amyopathic dermatomyositis (dermatomyositis siné myositis): a missing link within the spectrum of the idiopathic inflammatory myopathies. J Am Acad Dermatol.

[36] Narang NS, Casciola-Rosen L, Li S, Chung L, Fiorentino DF (2015). Cutaneous ulceration in dermatomyositis: Association with anti-melanoma differentiation-associated gene 5 antibodies and interstitial lung disease. Arthritis Care Res (Hoboken)..

[37] Fiorentino D, Chung L, Zwerner J, Rosen A, Casciola-Rosen L (2011). The mucocutaneous and systemic phenotype of dermatomyositis patients with antibodies to MDA5 (CADM-140): a retrospective study. J Am Acad Dermatol.

[38] Kumakura S, Murakawa Y (2014). Clinical characteristics and treatment outcomes of autoimmune-associated hemophagocytic syndrome in adults. Arthritis Rheumatol.

[39] Yamashita H, Matsuki Y, Shimizu A, Mochizuki M, Takahashi Y, Kano T, Mimori A (2013). Hemophagocytic lymphohistiocytosis complicated by central nervous system lesions in a patient with dermatomyositis: a case presentation and literature review. Mod Rheumatol.

[40] Poddighe D, Cavagna L, Brazzelli V, Bruni P, Marseglia GL (2014). A hyper-ferritinemia syndrome evolving in recurrent macrophage activation syndrome, as an onset of amyopathic juvenile dermatomyositis: a challenging clinical case in light of the current diagnostic criteria. Autoimmun Rev.

[41] Muro Y, Sugiura K, Akiyama M (2013). Limitations of a single-point evaluation of anti-MDA5 antibody, ferritin, and IL-18 in predicting the prognosis of interstitial lung disease with anti-MDA5 antibody-positive dermatomyositis. Clin Rheumatol.

[42] Koga T, Fujikawa K, Horai Y, Okada A, Kawashiri S-Y, Iwamoto N, Suzuki T, Nakashima Y, Tamai M, Arima K, Yamasaki S, Nakamura H, Origuchi T, Hamaguchi Y, Fujimoto M, Ishimatsu Y, Mukae H, Kuwana M, Kohno S, Eguchi K, Aoyagi K, Kawakami A (2012). The diagnostic utility of anti-melanoma differentiation-associated gene 5 antibody testing for predicting the prognosis of Japanese patients with DM. Rheumatology (Oxford).

[43] Nakashima R, Imura Y, Kobayashi S, Yukawa N, Yoshifuji H, Nojima T, Kawabata D, Ohmura K, Usui T, Fujii T, Okawa K, Mimori T (2010). The RIG-I-like receptor IFIH1/MDA5 is a dermatomyositis-specific autoantigen identified by the anti-CADM-140 antibody. Rheumatology (Oxford).

[44] Chen Z, Cao M, Plana MN, Liang J, Cai H, Kuwana M, Sun L (2013). Utility of anti-melanoma differentiation-associated gene 5 antibody measurement in identifying patients with dermatomyositis and a high risk for developing rapidly progressive interstitial lung disease: a review of the literature and a meta-analysis. Arthritis Care Res (Hoboken).

[45] Labrador-Horrillo M, Martinez MA, Selva-O’Callaghan A, Trallero-Araguas E, Balada E, Vilardell-Tarres M, Juárez C (2014). Anti-MDA5 antibodies in a large Mediterranean population of adults with dermatomyositis. J Immunol Res.

[46] Ceribelli A, Fredi M, Taraborelli M, Cavazzana I, Tincani A, Selmi C, Chan JYF, Chan EKL, Satoh M, Franceschini F (2014). Prevalence and clinical significance of anti-MDA5 antibodies in European patients with polymyositis/dermatomyositis. Clin Exp Rheumatol.

